# Microbiota: Not Just a Gut Feeling

**DOI:** 10.3390/jcm11206180

**Published:** 2022-10-20

**Authors:** Marcello Candelli

**Affiliations:** 1 Fondazione Policlinico Universitario Policlinico Gemelli—IRCCS, 00168 Rome, Italy; marcello.candelli@policlinicogemelli.it; 2 Department of Emergency, Anesthesiological and Reanimation Sciences, Catholic University of the Sacred Heart, 00168 Rome, Italy

Every year, an increasing number of scientific papers are published on the gut microbiome. According to PubMed, this number rocketed from about 500 publications in 2010 to over 10,000 in 2020, with a continued growth trend in subsequent years. One explanation for this incredible research interest is the new molecular and proteonomic techniques that facilitate the study of the complex components of the microbiome, and the now familiar concept that the gut microbiota is not a simple collection of microorganisms, but a true organ that has complex relationships and local and systemic impacts. This has allowed us to study the effects of the microbiota on extraintestinal diseases such as Alzheimer’s disease (AD), Parkinson’s disease (PD), coronary heart disease (CHD), and pancreatic diseases. Physiologically, the human microbiota consists largely (90%) of *Firmicutes* and *Bacteriodetes* Phila, with the remainder consisting of *Actinobacteria*, *Proteobacteria*, *Fusobacteria*, *Cyanobacteria*, and *Verrucomicrobia*. In many diseases, differences in the typical components of the microbiota have been associated with an increase or decrease in the abundance of one or more of the described Phila. Neurodegenerative diseases were among the first non-gastrointestinal diseases for which a link to the gut microbiota was sought. It has been known for decades that numerous bidirectional connections exist between the brain and the gut; this is known as the brain–gut axis, in which the gut microbiota plays a key role. The connections between the two organs occur through the nervous system (via the vagus nerve and enteric nervous system), the immune system, the endocrine system (hypothalamic–pituitary–adrenal axis), and the circulatory system with hormonal mediators, cytokines, and neurotransmitters [1,2]. For example, patients with PD have a higher abundance of the genera *Butyricimonas*, *Robinsoniella*, and *Flavonifractor*, compared with control subjects. The species *Akkermansia muciniphila* (*A. muciniphila*), *Eubacterium biforme*, and *Parabacteroides merdae* (*P. merdae*) are particularly abundant in patients with PD, whereas *Faecalibacterium prausnitzii*, *Ruminococcus albus*, and *Blautia faecis* are most abundant in healthy control subjects [3]. *A. muciniphila* and *P. merdae* are associated with the degradation of the mucus layer and intestinal mucins, the inflammation of the colon (found in over 80% of patients with PD), increased intestinal permeability, and the development of leaky gut. These alterations lead to an increase in endotoxinemia and oxidative stress, which in turn result in an increase in the misfolding of alpha-synuclein (α-syn) in the brain of PD patients [3]. Another mechanism for intestinal mucosal damage is related to the decreased number of species producing the short-chain fatty acid (SCFA) butyrate, which is essential for intestinal mucosal integrity [1,2,3]. The breakdown of mucins leads to increased water reabsorption, the production of dehydrated stool, and constipation. The latter typical symptom of PD could, therefore, be due not only to the motor changes typical of the disease, but also to intestinal dysbiosis [3]. There is also increasing evidence that PD may have an intestinal origin, and that α-Syn can travel from the ganglia of the enteric nervous system to the brain via the vagus nerve. Animal studies and the protective effect of vagotomy on the occurrence of PD seem to confirm this hypothesis [1]. It is also extremely interesting to note how, in mouse models of PD, the transplantation of feces from healthy mice resulted in the improvement of motor dysfunction, an increase in neurotransmitters of the striated nucleus, and a decrease in the inflammatory component of cerebral glia via the TLR4/TNF-α pathway [2]. Numerous studies have also been conducted on the relationships between the gut microbiota and Alzheimer’s disease (AD). The basic mechanisms of CNS–gut interaction are the same as described for PD, but in the case of AD, glial inflammation plays the main role [4]. Dysbiosis has also been observed in AD, with a loss of normal balance between the different components of the microbiota, an increase in potentially pathogenic genera such as *Klebsiella*, *Escherichia*, *Shigella*, *Proteus*, and *Clostridioides*, and a decrease in species associated with anti-inflammatory activity, such as *Lactobacillus* and *Bifidobacterium*. In addition, a decrease in the diversity of species present was observed. This affects the inflammatory burden, as there are fewer proinflammatory molecules such as SFCA and gamma-aminobutyric acid (GABA), but there is also an increase in toxic molecules such as lipopolysaccharide (LPS) and bacterial amyloid proteins. In addition, LPS derived from intestinal bacteria has been found in the brains of AD patients. It is hypothesized that LPS and amyloid proteins can enter the brain from the gut via two pathways: either retrograde axonal transport via the vagus nerve, as previously described for PD, or via the bloodstream once gut permeability and the blood–brain barrier are disrupted [4,5]. The link between the activation of cerebral astrocytes (major components of glia) and the microbiota is thought to result from the metabolism of dietary tryptophan by intestinal bacteria that form indole-3-aldehyde and indole-3-propionic acid. These molecules bind to aryl hydrocarbon receptors (AHR) located on the cell membrane of astrocytes and regulate their activation and local inflammation together with gamma interferon. Another mechanism of the inflammatory activation of astrocytes by the microbiome involves bacterial products such as LPS, peptidoglycans, and PAMPs (pathogen-associated molecular patterns) which, in the case of dysbiosis with consequent alteration of the intestinal barrier and blood–brain barrier, can reach cerebral glia and activate astrocytes via TLR4 (Tool-like receptor 4) [4,5]. Alteration of the gut microbiota has also been linked to the cardiovascular system, both directly and indirectly, through the induction of risk factors (such as atherosclerosis and increased blood pressure or the alteration of lipid metabolism) [6,7,8,9,10]. Numerous animal models have shown how the presence of dysbiosis can affect blood pressure through the production of SCFA and, in particular, propionate, which has a direct and dose-dependent effect on arterial dilation, increases the release of renin, and lowers blood pressure by downregulating genes involved in the expression of early growth response protein-1 (Egr1). The activation of Egr1 can also cause myocardial hypertrophy, induce cardiac fibrosis, and increase systemic inflammation [6]. Bacteria present in the intestine also metabolize dietary phosphatidylcholine to trimethylamine N-oxide (TMAO), the plasma level of which directly correlates with hypertension and the development of arterial stiffness and atherosclerosis. An increase in intestinal lactobacilli through oral supplementation can reduce plasma TMAO levels and, thus, blood pressure. In addition, the microbiota also intervenes by metabolizing bile acids in the intestine and interacting with two bile acid receptors—the nuclear receptor farnesoid X receptor (FXR) and the cell membrane receptor Takeda G protein-coupled receptor 5 (TGR5)—leading to an increase in plasma low-density lipoproteins (one of the major cardiovascular risk factors). Finally, a specific microbiota composition has been associated with ischemic heart disease. In patients with myocardial infarction, an excess of proinflammatory genera such as *Enterobacteriaceae* and *Streptococcus* spp. was observed, as well as a reversal of the normal ratio between *Firmicutes* and *Bacteroidetes*. A recent study also showed that patients with acute myocardial infarction had a marked increase in a particular bacterial species, *Selenomonadales*, and selenium-based compounds compared with a group of healthy subjects [8,9]. However, all these positive findings should not make us forget that sometimes unexpected and negative results can be obtained. By changing the target organ, we must indeed point out that even a seemingly simple and safe therapy that interferes with the alteration of the microbiota, such as the administration of probiotics, can be rather harmful. Although an inflammatory state and altered intestinal permeability, likely due to dysbiosis, have been described in pancreatitis, the administration of probiotics resulted in increased mortality in a clinical trial [10]. Although these results have since been reevaluated and attributed to the lactic acid fermentation of dietary carbohydrates, caution and patient safety must always be paramount in such a complicated area with multiple and systemic interactions. Unfortunately, although there are hundreds of publications on this topic, the impact on clinical practice is still minimal. The therapies derived from research on the microbiota that are accepted by guidelines in the field of gastroenterology are fecal transplantation for antibiotic-resistant *C. difficile* infections, the use of probiotics in pouchitis, in the prevention of necrotizing enterocolitis, and in the prevention of *C. difficile* infections during antibiotic therapy. In contrast, we are still waiting for therapeutic implications for all non-gastrointestinal pathologies associated with intestinal dysbiosis. However, there are promising studies showing that both a low-fat diet and the administration of probiotics reduce the risk of developing AD and cognitive disorders in both animal models and human epidemiological studies [4]. However, understanding the molecular mechanisms of dysbiosis could lead to the development of new therapies based on nutritional aspects, the use of pro-, pre-, and antibiotics, and fecal transplants. The field of research is wide and open. Prospects for therapeutic alternatives that can be combined with existing ones are very promising, and several trials in various phases of clinical testing (some in phase 3) will be completed in coming years to determine the effects of altering the microbiota in many non-gastrointestinal diseases [4].
